# Clinical evaluation of super-responders vs. non-responders to CGRP(-receptor) monoclonal antibodies: a real-world experience

**DOI:** 10.1186/s10194-023-01552-x

**Published:** 2023-02-27

**Authors:** Bianca Raffaelli, Mira Fitzek, Lucas H. Overeem, Elisabeth Storch, Maria Terhart, Uwe Reuter

**Affiliations:** 1 Department of Neurology, Charité – Universitätsmedizin Berlin, Charitéplatz 1, Berlin, 10117 Germany; 2 Clinician Scientist Program, Berlin Institute of Health at Charité (BIH), Berlin, Germany; 3 Universitätsmedizin Greifswald, Greifswald, Germany

**Keywords:** Nonresponder, CGRP-mAb, Predictors

## Abstract

**Background:**

Clinical trials and real-world studies revealed a spectrum of response to CGRP(-receptor) monoclonal antibodies (mAbs) in migraine prophylaxis, ranging from no effect at all to total migraine freedom. In this study, we aimed to compare clinical characteristics between super-responders (SR) and non-responders (NR) to CGRP(-receptor) mAbs.

**Methods:**

We performed a retrospective cohort study at the Headache Center, Charité – Universitätsmedizin Berlin. The definition of super-response was a ≥ 75% reduction in monthly headache days (MHD) in the third month after treatment initiation compared to the month prior to treatment begin (baseline). Non-response was defined as ≤ 25% reduction in MHD after three months of treatment with a CGRP-receptor mAb and subsequent three months of treatment with CGRP mAb, or vice versa. We collected demographic data, migraine disease characteristics, migraine symptoms during the attacks in both study groups (SR/NR) as well as the general medical history. SR and NR were compared using Chi-square test for categorical variables, and t-test for continuous variables.

**Results:**

Between November 2018 and June 2022, *n* = 260 patients with migraine received preventive treatment with CGRP(-receptor) mAbs and provided complete headache documentation for the baseline phase and the third treatment month. Among those, we identified *n* = 29 SR (11%) and *n* = 26 NR (10%). SR reported more often especially vomiting (SR *n* = 12/25, 48% vs. NR *n* = 4/22, 18%; *p* = 0.031) and typical migraine characteristics such as unilateral localization, pulsating character, photophobia and nausea. A subjective good response to triptans was significantly higher in SR (*n* = 26/29, 90%) than in NR (*n* = 15/25, 60%, *p* = 0.010). NR suffered more frequently from chronic migraine (NR *n* = 24/26, 92% vs. SR *n* = 15/29, 52%; *p* = 0.001), medication overuse headache (NR *n* = 14/24, 58% versus SR *n* = 8/29, 28%; *p* = 0.024), and concomitant depression (NR *n* = 17/26, 65% vs. SR *n* = 8/29, 28%; *p* = 0.005).

**Conclusion:**

Several clinical parameters differ between SR and NR to prophylactic CGRP(-R) mAbs. A thorough clinical evaluation prior to treatment initiation might help to achieve a more personalized management in patients with migraine.

**Supplementary Information:**

The online version contains supplementary material available at 10.1186/s10194-023-01552-x.

## Background

Monoclonal antibodies (mAb) against Calcitonin Gene-Related Peptide (CGRP) and the CGRP-receptor (CGRP-R) are specific substances for the prophylactic treatment of migraine [1]. Clinical trials have demonstrated the efficacy, safety and favorable tolerability of CGRP(-R) mAbs in patients with episodic migraine (EM) and chronic migraine (CM) [2].

In the pivotal registration trials for the CGRP-R mAb erenumab, up to 50% of patients with EM and 41% of patients with CM reported a reduction in monthly migraine days (MMD) from baseline of at least 50% [3–5]. Studies with the CGRP mAbs galcanezumab and fremanezumab reached 50% responder rates of up to 62% in EM [6–8] and 41% in CM [9, 10]. Of note, trial designs and statistical analyses are not identical between studies and therefore numerical differences exist in the endpoint determination [3–8]. Even in patients with numerous unsuccessful prior preventive treatment attempts, CGRP(-R) mAbs led to a ≥ 50% response in one-third of cases [11–13]. Despite these favorable results, up to 20% of patients had no improvement with CGRP(-R) mAbs, showing no change or even an increase in MMD during these trials [14]. In contrast, a subgroup of trial participants showed an exceptionally good treatment response: Up to 39% of EM patients achieved a ≥ 75% decrease in MMD and up to 16% were completely migraine-free during the selected observation period [7, 14].

Real-world data with the use of CGRP(-R) mAbs in clinical practice show similar findings. About half of CM patients have a 50% reduction of MMD after three months of treatment [15, 16]. At the same time, up to one-third of patients do not respond to mAbs treatment, while 20% have a very high benefit with a reduction of ≥ 75% in MMD along a tremendous improvement of quality of life.

Despite several attempts, clear predictors of response could not be determined in the registration trials. In a few real-world studies, unilateral pain localization, and triptan responsiveness were positively associated with a better treatment response [17–21]. On the contrary, psychiatric comorbidities, a long disease duration and a high number of previously failed preventive treatments were associated with a lower response [17, 18, 22–26]. Additional file 1 provides an overview of current literature on predictors of response to treatment with CGRP(-R)-mAbs.

However, patients who have no improvement with mAbs prophylaxis and patients who respond exceptionally well have not been characterized to date. Clinical differences between non-responders (NR) and super-responders (SR) could help to facilitate a better tailored management of patients with migraine. In this study, we aim to perform a clinical evaluation of absolute NR and SR to CGRP(-R) mAbs and to compare clinical characteristics between these two groups.

## Methods

### Study design and participants

This was a retrospective cohort study at the tertiary Headache Center, Charité – Universitätsmedizin Berlin, Germany. We screened the electronic charts of all patients with migraine who received prophylactic treatment with CGRP(-R) mAbs between November 2018 and June 2022. Due to reimbursement regulations in Germany at the time of this study, the prerequisites for mAb treatment were treatment failure or intolerable adverse events with all first-line preventives (beta-blockers, topiramate, flunarizine, amitriptyline and for CM also OnabotulinumtoxinA) or contraindications to those [27–29].

Super-Responder (SR) and Non-Responder (NR) were identified according to the following definitions:

- SR: Patients with ≥ 75% reduction of monthly headache days (MHD) in the third month after mAb treatment initiation versus baseline (i.e. the four-week period prior to the first mAb injection or, if available, the average number of MHD of the last three months prior to the first mAb treatment).
- NR: Patients who received a CGRP-R-mAb (erenumab) and subsequently a CGRP-mAb (galcanezumab or fremanezumab), or vice versa, and had a reduction in MHD of ≤ 25% between baseline and the third month of treatment with both mAb classes.

Headache data was assessed using standardized electronic or paper headache diaries that the patients routinely bring to every appointment. A month was defined as 28-day period, a headache day as any calendar day with a documented headache episode. When headache diaries were missing, we used the physician’s documentation in the electronic patient chart. Patients without any headache documentation for the period of interest were excluded from this analysis. Further exclusion criteria were: mAb treatment duration of less than three months, and prior participation in a registration trial with CGRP(-R) mAbs.

### Evaluation of Super-Responders (SR) and Non-Responders (NR)

In the groups of SR and NR, we recorded basic demographic data, migraine history and characteristics as well as general medical history (Table 1).

**Table 1:** Collected parameters in Super-Responders (SR) and Non-Responders (NR) with the respective answer possibilities

Parameter	Answer possibilities
**Demographic data**	
Age at initiation of treatment with CGRP(-R) mAbs	Age in years
Sex	Female/Male
**Migraine: Disease characteristics**	
Disease duration	Time since migraine onset in years
Type of migraine prior to mAb treatment	Episodic/Chronic
Aura	Yes/No
Monthly headache days (MHD) at baseline	Number of days ≤ 28
Monthly migraine days (MMD) at baseline	Number of days ≤ 28
Non-migraine days (NMD) at baseline	Number of days ≤ 28
**Migraine: Symptoms during the attacks**	
Average untreated attack	Duration in hours
Average untreated pain intensity	Numeric analogue scale 0–10
Unilateral location	Yes/No
Pulsating quality	Yes/No
Aggravation by physical activity	Yes/No
Nausea	Yes/No
Vomiting	Yes/No
Photophobia	Yes/No
Phonophobia	Yes/No
**Migraine: Acute and preventive treatment**	
Use of triptans as acute treatment	Yes/No
Subjective good response to triptans	Yes/No
Number of prior preventive substances	Number
For NR: Following treatment strategy	Name of medication
**General medical history**	
Concomitant depression	Yes/No
Concomitant anxiety disorder	Yes/No
Concomitant other chronic pain condition	Yes/No
Concomitant medication overuse headache (MOH)	Yes/No

A monthly migraine day (MMD) was defined as any calendar day with a headache fulfilling the International Classification of Headache Disorders-3 (ICHD-3) of migraine or probable migraine [30]. Non-migraine days (NMD) were defined as headache days that did not fulfill this criterion (NMD = MHD – MMD). Baseline period: For migraine symptoms during attacks, each item was considered positive if the majority of attacks displayed the respective feature.

### Statistical analysis

Statistical analysis was performed using IBM SPSS Statistics, version 27. We used descriptive statistics to summarize demographic and anamnestic data (frequencies and percentages for categorical variables, mean ± standard deviation for numerical variables). Categorical variables were compared between groups using the Chi-square test, and continuous variables by t-test. A two-tailed *p* value < 0.05 was considered statistically significant. Due to the exploratory approach of the study, data were not corrected for multiple testing. Numbers and percentages of missing values are provided for each variable.

## Results

Between November 2018 and June 2022, *n* = 359 patients received at least one CGRP(-R) mAb as preventive treatment for migraine. Headache documentation for baseline and the third mAb treatment month was available for *n* = 260 patients. Of these, *n* = 29 (11%) and *n* = 26 (10%) met the predefined criteria of a SR or NR, respectively.

NR and SR did not differ significantly with respect to sex, age at initiation of mAb therapy, or duration of migraine in years (Table 2). In both NR and SR groups, erenumab was the most frequent mAb of first choice (Table 2). The class of the first used mAb (CGRP-R or CGRP mAb) did not differ between NR and SR (NR CGRP-R mAb *n* = 14/26, 54% vs. SR CGRP-R-mAb *n* = 12/29, 41%; *p* = 0.35).).

**Table 2:** Demographic characteristics of Non-Responders and Super-Responders to CGRP(-R) mAbs

	**Non-Responders**	**MD**	**Super-Responders**	**MD**
**n**	26		29	
**Female, n (%)**	22 (85%)	-	22 (76%)	-
**Age in years at first** **administration, mean ± SD**	50.5 ± 10.8	-	49.0 ± 10.7	-
**Years lived with migraine,** **mean ± SD**	31.5 ± 19.1	4	30.2 ± 13.1	9
**First mAb: n (%)**	Erenumab: 14 (54%) Fremanezumab: 4 (15%) Galcanezumab: 8 (31%)		Erenumab: 12 (41%) Fremanezumab: 10 (35%) Galcanezumab: 7 (24%)	-
**Second mAb: n (%)**	Erenumab: 12 (46%) Fremanezumab: 2 (8%) Galcanezumab: 12 (46%)		-	-

*Abbr.: SD* Standard deviation, *MD* Missing data

### Headache characteristics

**Fig. 1:**
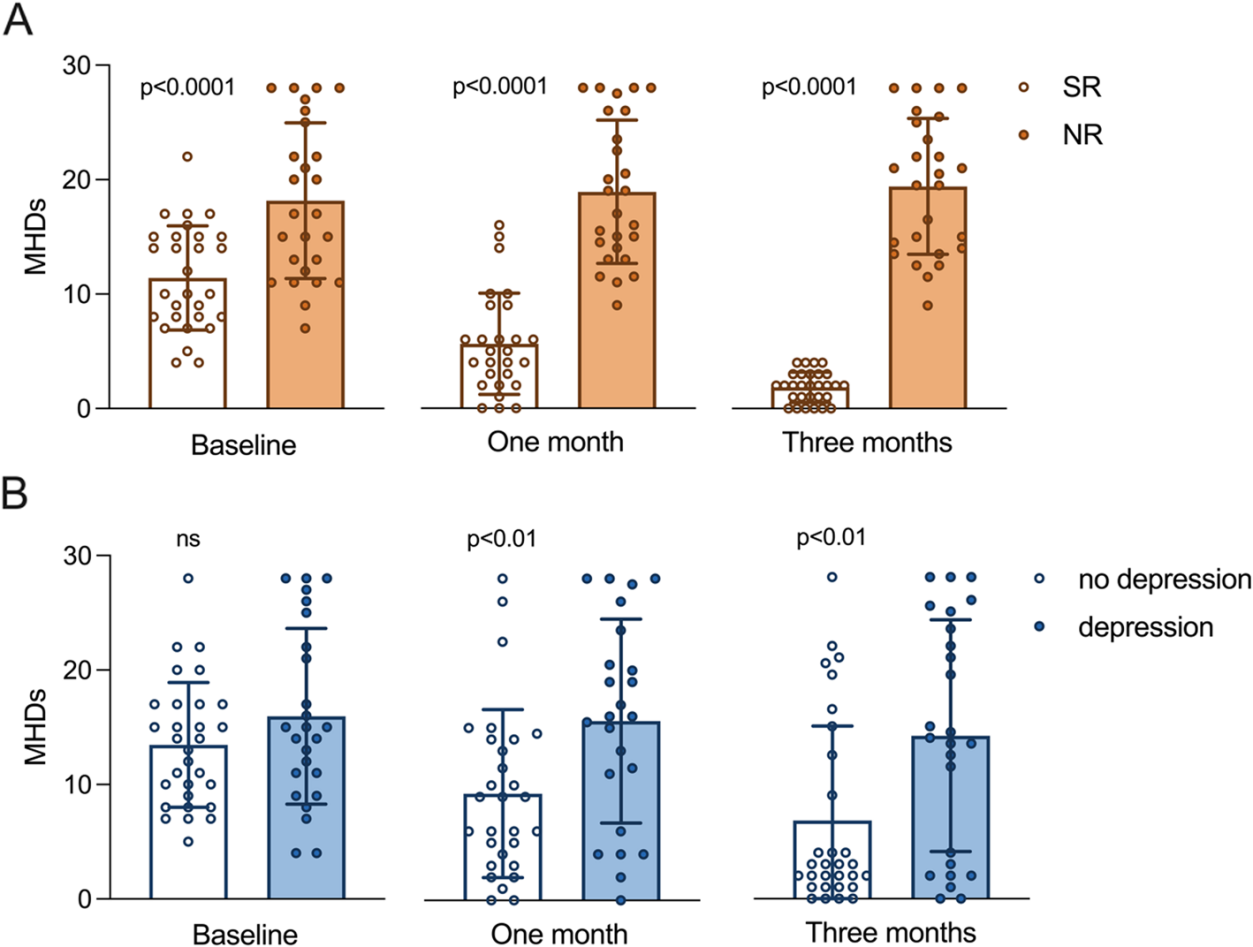
Pattern of monthly headache days over three months of CGRP-mAb therapy. **A** In Super-Responders and Non-Responders. **B** In patients with and without concomitant depression. NR were significantly more likely to have concomitant depression

NR suffered significantly more often from CM than SR (NR *n* = 24/26, 92% vs. SR *n* = 15/29, 52%; *p* = 0.001). Accordingly, the number of MHD (NR 18.2 ± 6.8 vs. SR 11.4 ± 4.5, t = 4.4, *p* < 0.001) and MMD (NR 14.1 ± 6.4 vs SR 10.6 ± 4.7, t = 2.2, *p* = 0.035) during baseline were significantly higher in NR than in SR (Fig. 1). NR suffered also from more NMD at baseline, although without statistical significance (NR 3.5 ± 6.7 vs. SR 0.8 ± 2.2, t = 1.98, *p* = 0.054). Typical migraine characteristics such as unilateral localization, pulsating character, photophobia, nausea or vomiting occurred numerically more frequently in SR than NR (Table 3), but statistical significance was only reached for the occurrence of vomiting (NR *n* = 4/22, 18% vs. SR *n* = 12/25, 48%; *p* = 0.031).

**Table 3:** Key headache baseline characteristics of Non-Responders and Super-Responders to CGRP(-R) mAbs

	**Non-Responders**	**MD**	**Super-Responders**	**MD**	***P* value**
**Number of MHDs, mean ± SD**	18.2 ± 6.8	-	11.4 ± 4.5	-	< 0.001*
**Number of MMDs, mean ± SD**	14.1 ± 6.4	4	10.6 ± 4.7	4	< 0.035*
**Chronic migraine, n (%)**	24 (92%)	-	15 (48%)	-	0.001*
**Aura, n (%)**	8 (15%)	-	13 (24%)	-	0.356
**Intensity 1–10, mean ± SD**	7.2 ± 1.9	1	7.0 ± 2.2	2	0.729
**Attack duration (h),** **mean ± SD**	44.2 ± 26.6	10	31.5 ± 28.7	7	0.172
**Unilateral headache, n (%)**	18 (72%)	1	25 (93%)	2	0.129
**Pulsating character, n (%)**	19 (76%)	1	26 (93%)	1	0.231
**Aggravation by physical activity, n (%)**	22 (100%)	4	25 (100%)	4	0.867
**Photophobia, n (%)**	21 (88%)	2	26 (96%)	2	0.503
**Phonophobia, n (%)**	20 (87%)	3	24 (89%)	2	0.818
**Nausea, n (%)**	21 (88%)	2	27 (100%)	2	0.166
**Vomitus, n (%)**	4 (18%)	4	12 (48%)	4	0.031*

*Abbr.: MHD* Monthly Headache Days, *MMD* Monthly Migraine Days, *SD* Standard deviation, *MD* Missing data

^*^ Statistically significant

### Migraine specific medication

Both patient groups used triptans as acute migraine medication (NR *n* = 25/26, 98%, and SR *n* = 29/29, 100%). However, SR were significantly more likely to report sufficient improvement of their acute migraine headache after triptan intake (*n* = 26/29, 90%) compared to NR (*n* = 15/25, 60%, *p* = 0.010). The number of pharmacological prophylactic treatment regimens prior to CGRP(-R) mAb therapy did not differ significantly between NR and SR (NR 4.9 ± 1.8 vs. SR 4.8 ± 2.2, *p* = 0.8).

### Comorbidities

NR suffered significantly more often from concomitant depression than SR (NR *n* = 17/26, 65% vs. SR *n* = 8/29, 28%; *p* = 0.005) (Fig. 1). Medication overuse headache (MOH) prior to treatment begin was also statistically significantly more frequent in NR than SR (NR *n* = 14/24, 58% versus SR *n* = 8/29, 28%; *p* = 0.024). Other comorbidities, such as anxiety disorders or other chronic pain disorders, did not differ significantly.

### Follow-up therapy in non-responders

**Fig. 2:**
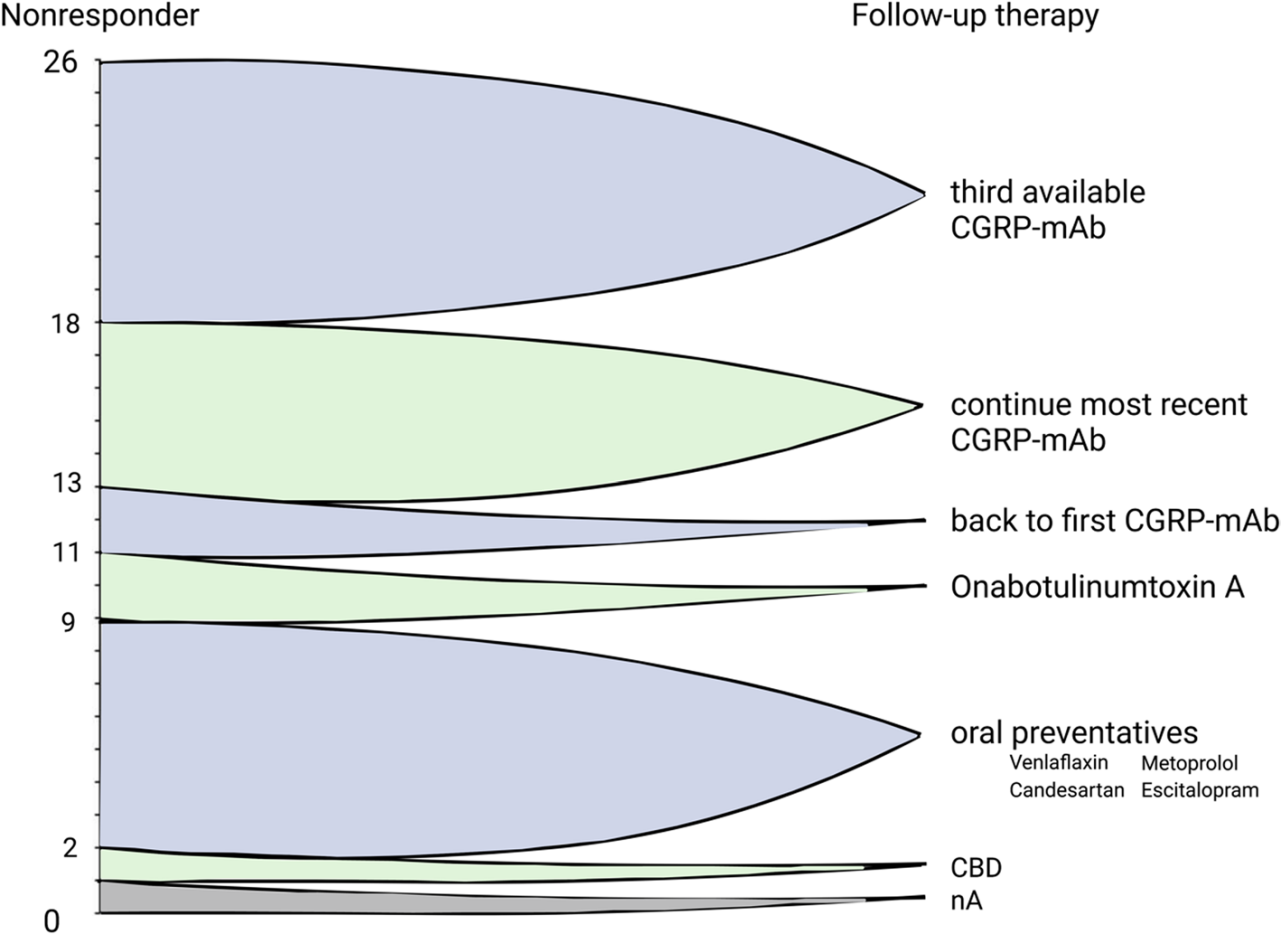
Follow-up therapy in Non-Responders after unsuccessful treatment with both CGRP mAb and CGRP-R mAb. The left Y-axis shows the number of individual NR (*n* = 26) and the right Y-axis the follow up therapy performed in these patients

In NR, therapy was switched to the third and at this time last available CGRP-(R) mAb in 31% of cases (*n* = 8/26). In 19% (*n* = 5/26), the most recent CGRP-(R) antibody therapy was continued due to subjective headache improvement, e.g. reported improvement in headache intensity or a better response to acute headache medication. Figure 2 shows the follow-up treatment strategies in NR.

## Discussion

In this retrospective real-world study, we identify clinical characteristics of super-responders (SR) and absolute non-responders (NR) to CGRP(-R) mAbs. Migraine attacks in SR were numerically more likely to present with typical migraine features such as unilateral localization, pulsating character, nausea, and vomiting than in NR, with only vomiting reaching statistical significance. Conversely, NR had twice as often chronic migraine (CM), medication-overuse headache (MOH) and suffered from depression in comparison to SR.

Due to regulatory requirements in Germany [27–29], treatment with CGRP(-R) mAbs at the time of this study was reimbursed only in patients with treatment-resistant migraine [31]. Response to CGRP(-R)-mAbs was shown to be better in patients with a lower number of unsuccessful prophylactic treatment attempts [16, 18, 25, 32], but even in this difficult-to-treat population, 11% of patients showed an excellent clinical response to CGRP(-R) mAbs with a ≥ 75% reduction in headache frequency. This is lower than in cohorts with less treatment-experienced patients [3–8] but still remarkable for patients who have lived with migraine for decades and failed all preventives of first choice. In this subgroup of patients, CGRP seems to be the driving force for migraine attack generation. Accordingly, in a Danish study, patients with an excellent response to erenumab were also highly susceptible to intravenous CGRP provocation, suggesting CGRP to be the key pathophysiological pathway in this clinical phenotype (“CGRP phenotype”) [33]. Migraine-specific acute medications such as triptans also act on the CGRP pathway [1]. Triptans target mainly the 5-HT_1B_ and 5-HT_1D_ receptor subtypes, located at meningeal vessels but also within the trigeminal nerve endings and trigeminal nucleus and suppresses the release of CGRP [34, 35]. Typical accompanying symptoms of migraine such as photophobia or vomiting are also mediated by CGRP and relieved by both triptans and CGRP(-R) mAbs [36, 37]. In the presented study, consistent with previous publications [20, 38], a good subjective response to acute therapy with triptans was reported significantly more often by SR. SR also reported more typical concomitant symptoms, in particular vomiting. Both findings are in line with the assumption that CGRP plays a central role in migraine initiation in this subgroup.

On the other side, in 10% of the patients neither CGRP nor CGRP(-R) mAbs demonstrated a clinical meaningful effect. This is less than previously reported for single CGRP(-R)-mAbs [3–8] and may be due to the fact that a certain proportion of NR to a single CGRP(R)-mAb respond to the change of mAb class. A recent German multicenter study showed that one-third of NR to the CGRP(-R)-mAb erenumab responded well when switched to a CGRP(-R)-mAb [39].

Previous studies revealed that the number of MHD correlates inversely with the likelihood of a positive treatment response to CGRP(-R) mAb [19, 32, 40–42]. In line with these observations, NR of the present study suffered significantly more often from CM and had a significantly higher frequency of MHD and MMD at baseline. Persistent activation of inflammatory signal pathways and neuronal sensitization play a key role in migraine progression and chronification, causing sustained neuronal hypersensitivity [43, 44]. While CGRP is one important mediator in the development of sensitization [45], several other neurotransmitters are involved, e.g. vasoactive intestinal peptide (VIP), and pituitary adenylate cyclase-activating polypeptide (PACAP) [46, 47]. It is possible that in clinical NR to CGRP(-R) mAbs, CGRP has a negligible pathophysiological role and other molecular pathways prevail. In line with this hypothesis, one-third of migraine patients do not develop migraine following the intravenous administration of CGRP (“non-CGRP phenotype”) [33, 48].

Apart from these pathophysiological considerations, psychopathological conditions are thought to act as migraine worsening factors [18]. Depression is a frequent comorbidity of migraine, particularly CM [49] and is associated with higher headache frequency [50]. Consistent with the clinical profile of patients with refractory migraine and treatment failure in CGRP-mAb proposed by Silvestro et al. [51], in our cohort, NR suffered significantly more frequently from concomitant depression than SR. Presence of comorbid depression is not only associated with poor treatment response to CGRP-mAb therapy, but was also shown to be a negative predictor of good treatment response to Onabotulinumtoxin A [52, 53]. In light of its negative association to prophylactic treatment response, more attention should be drawn to concomitant depression in migraine focusing on the evaluation of potential need for psychotherapeutic or pharmacotherapeutic treatment. Selected patient may benefit from combination therapy of antidepressants and CGRP-mAb and multimodal treatment concepts should be strived for in this burdened patient group.

Another frequent comorbidity in NR was MOH. Medication overuse is associated with poor response to acute medications [54], contributes the process to chronicity as well as treatment refractivity of migraine [55], and is therefore considered to be a prognostic factor for poor response to preventive treatment [26, 45]. Our results are in line with previous findings of Silvestro et al [51] and Ornello et al [56] who demonstrated greater medication overuse in patients with therapy resistant migraine to CGRP(-R) mAb therapy. However, several studies have demonstrated efficacy of CGRP(-R) mAb in MO with a remission von MO to non-MO [57]. Therefore, MO alone may not be the determining factor for the lack of response to therapy, but rather a combination of different clinical features and comorbidities. In patients with MOH and inadequate response to CGRP(-R) mAbs, withdrawal can be attempted, as central sensitization can successfully improve after detoxification [58] and studies suggest better response to preventive treatment after withdrawal [57].

Evidence-based recommendations regarding CGRP(-R) mAb non-response after multiple other preventatives are lacking [59]. Even though both classes of CGRP(-R) mAb had already been tested unsuccessfully, the most frequent change in our group of NR was to the third and last available CGRP-mAb. The reason behind this clinical decision may be the slightly different pharmacokinetics and pharmacodynamics of galcanezumab and fremanezumab [2] but, above all, the lack of alternatives in patients who had already tried all treatments of first choice unsuccessfully. Presumably in the absence of alternatives, a non-negligible proportion of NR also switched back to oral prophylactics or Onabotulinumtoxin A, although these had not shown sufficient efficacy previously. The evaluation of these treatment strategies was not the subject of this investigation and should be assessed in future studies with a larger sample size.

Our study was designed as a retrospective analysis of data from our headache outpatient clinic, so no causal relationships can be drawn. Our aim was to describe the characteristics of the two groups of SR and NR in an initially exploratory approach. In the next step, a longitudinal study to verify those results would be necessary. The rigorously chosen criteria for SR and NR allow us to describe characteristics that apply for these two specific groups of patients. However, the strict inclusion criteria result in a limited sample size for the groups of interest. Some of the described trends could possibly reach statistical significance if the groups were larger. Future multicenter studies should provide for larger cohort to ensure a higher statistical power. Further limitations include some missing data of headache characteristics, which can cause bias in the estimation of results.

## Conclusion

The presence of chronic migraine, high headache frequency at baseline, medication overuse, concomitant depression, and subjective poor response to acute therapy with triptans are typical features of non-responders to CGRP-targeted treatment. Conversely, a lower headache frequency prior to treatment begin, good response to triptans and pronounced vegetative symptoms such as vomiting occur more often in super-responders. This work is in line with the existing evidence on treatment response in patients treated with CGRP(-R) mAbs but expands into a highly treatment-resistant cohort. An accurate clinical evaluation before starting treatment with CGRP(-R) mAbs might help to achieve a more tailored therapeutic management in patients with migraine.

## Supplementary Information

**Additional file 1.** Summary of current literature on predictors of good clinical response to treatment with CGRP(-R)-mAbs.

## Data Availability

The datasets used and/or analysed during the current study are available from the corresponding author on reasonable request.

## Abbreviations

CM: Chronic migraine
CGRP: Calcitonin Gene-Related Peptide
CGRP-R: Calcitonin Gene-Related Peptide-receptor
EM: Episodic migraine
ICHD-3: International Classification of Headache Disorders 3
mAb: Monoclonal antibody
MHD: Monthly headache days
MD: Missing data
MMD: Monthly migraine days
MOH: Medication overuse headache
NMD: Non-migraine days
NR: Non-responder
SD: Standard deviation
SR: Super-responder
