# Supplementary material for: Clinical evaluation of super-responders vs. non-responders to CGRP(-receptor) monoclonal antibodies: a real-world experience
Source: J Headache Pain. 2023 Feb 27;24(1):16. doi: 10.1186/s10194-023-01552-x (PMC9969617; doi:10.1186/s10194-023-01552-x)
Supplement: Supplementary file 1 — Additional file 1. Summary of current literature on predictors of good clinical response to treatment with CGRP(-R)-mAbs. [file 10194_2023_1552_MOESM1_ESM.docx]

**Additional file 1:** Summary of current literature on predictors of good clinical response to treatment with CGRP(-R)-mAbs.

| **Antibody** | **N** | **EM/CM** | **Definition of response** | **Predictors of good response** | **Ref.** |
| --- | --- | --- | --- | --- | --- |
| Galcanezumab | 156 | CM | ≥50% reduction in MHD during three consecutive months (months 1-3) | - Lower body mass index - Unilateral pain localisation - Good response to triptans - MO at baseline - Lower number of prior preventive treatments | 16 |
| Erenumab | 103 | (HF)EM | ≥50% reduction in MMD at weeks 9-12 | - Unilateral pain localisation | 17 |
|  | 269 | CM | ≥50% reduction in MHD at weeks 9-12 | - Higher number of MHD at baseline - Dopaminergic symptoms^a^ - Lack of psychiatric comorbidities | 17 |
| Erenumab | 57 | (HF)EM | ≥50% reduction in MMD at weeks 45-48 | - Higher HIT-6 score at baseline - Cutaneous allodynia - Lower number of prior treatment failures | 18 |
|  | 164 | CM | ≥50% reduction in MHD at weeks 45-48 | - Male sex - Higher number of MHD at baseline - Higher HIT-6 score at baseline - Dopaminergic symptoms* - Lower number of prior treatment failures - Lack of psychiatric comorbidities - Good response to triptans - Lower monthly analgesic intake | 18 |
| Erenumab/  Galcanezumab/  Fremanezumab | 208 | (HF)EM | ≥ 50% reduction in MMD at weeks 21-24 | - Unilateral pain + unilateral cranial autonomic symptoms | 19 |
|  | 656 | CM | ≥ 50% reduction in MMD at weeks 21-24 | - Unilateral cranial autonomic symptoms - Unilateral pain + unilateral cranial autonomic symptoms - Unilateral pain + allodynia - Lower BMI - Lower MHD at baseline | 19 |
| Erenumab | 105 | (HF)EM/CM | ≥ 50% mean reduction in MMD at months 4-6 | - Good response to triptans | 20 |
| Erenumab / Fremanezumab | 123 | (HF)EM/CM | ≥ 50% reduction in MMD at weeks 9-12 | - Unilateral pain localization - Higher HIT-6 score at baseline - Positive family history - Lower mean blood flow velocity in the right MCA | 21 |
| Erenumab | 70 | CM | ≥30% reduction in MHD at weeks 9-12 | - Shorter disease duration | 22 |
| Erenumab | 75 | CM | ≥ 50% reduction in MHD at month 12 | - Lower prevalence of personality disorders belonging to Cluster C^b^ - Lower prevalence of anxiety disorders - Lower number of “at least serious” current stressors | 23 |
| Erenumab/  Galcanezumab/  Fremanezumab | 203 | CM | ≥ 50% reduction in MMD at month 1 and 6 | - Lower number of MMD at baseline - Shorter duration of chronicization - Less total number of analgesics | 24 |
| Erenumab | 110 | EM/CM | ≥ 50% reduction in MMD at weeks 9-12  ≥ 75% reduction in MMD at weeks 9-12 | - Lower body mass index - Lower number of prior preventive treatments - Older age at migraine onset - Lower number of prior preventive treatments - Higher MIDAS score at baseline | 25 |
| Erenumab | 111 | CM with MO | ≥30% reduction in MHD at months 10-12 | - Male sex - Lower number of prior preventive treatments - Longer MO duration - Lower number of MHD at baseline - Lower number of AMD at baseline - Lower MIDAS score at baseline | 31 |
| Erenumab | 172 | EM/CM | ≥ 50% reduction in MMD at weeks 9-12 | - EM - Good response to triptans - Absence of TTH | 37 |
| Erenumab | 98 | CM | ≥30% reduction in MHD at weeks 9-12 or  ≥50% reduction in severe headache days at weeks 9-12 | - Lower number of MHD at baseline | 41 |
| Erenumab | 89 | CM | ≥ 50% reduction in MMD after at least one of the first 3 doses | - Lower number of MMD at baseline - Lower number of AMD at baseline | 55 |
| Fremanezumab | 17 | (HF)EM | ≥ 50% reduction in MMDs at weeks 9-12 | - Lower monthly analgesic intake - More frequent use of monthly dosing regimen | 59 |
|  | 36 | CM | ≥ 50% reduction in MHDs at weeks 9-12 | - Younger age - Shorter MO duration - More frequent use of monthly dosing regimen - Lower HIT-6 scores at baseline | 59 |

Abbr: AMD = monthly days with intake of acute medication, CM = chronic migraine, EM = episodic migraine, HF = high frequency, HIT-6 = Headache Impact Test-6, MCA = middle cerebral artery, MHD = monthly headache days, MIDAS = Migraine Disability Assessment, MMD = monthly migraine days, MO = Medication overuse, N = total number of patients, Ref = reference, TTH = tension type headache. ^a^Dopaminergic symptoms = yawning, somnolence, nausea, or vomiting during the prodromes, headache stage, or postdromes. ^b^Cluster C personality disorders = avoidant, dependent, and obsessive-compulsive. The reference numbers correspond to the main manuscript.
